# Evolutionary history of rat-borne *Bartonella*: the importance of commensal rats in the dissemination of bacterial infections globally

**DOI:** 10.1002/ece3.702

**Published:** 2013-08-06

**Authors:** David T S Hayman, Katherine D McDonald, Michael Y Kosoy

**Affiliations:** 1 Department of Biology, Colorado State UniversityFort Collins, Colorado; 2 Department of Biology, University of FloridaGainesville, Florida 32611; 3 Division of Vector-Borne Diseases, Centers for Disease Control and PreventionFort Collins, Colorado

**Keywords:** Bayesian inference, emerging pathogens, invasive species, phylogeography, *Rattus*, reservoir host

## Abstract

Emerging pathogens that originate from invasive species have caused numerous significant epidemics. Some bacteria of genus *Bartonella* are rodent-borne pathogens that can cause disease in humans and animals alike. We analyzed *glt*A sequences of 191 strains of rat-associated bartonellae from 29 rodent species from 17 countries to test the hypotheses that this bacterial complex evolved and diversified in Southeast Asia before being disseminated by commensal rats *Rattus rattus* (black rat) and *Rattus norvegicus* (Norway rat) to other parts of the globe. The analysis suggests that there have been numerous dispersal events within Asia and introductions from Asia to other regions, with six major clades containing Southeast Asian isolates that appear to have been dispersed globally. Phylogeographic analyses support the hypotheses that these bacteria originated in Southeast Asia and commensal rodents (*R. rattus* and *R. norvegicus*) play key roles in the evolution and dissemination of this *Bartonella* complex throughout the world.

## Introduction

The ecology and evolution of infectious agents are critical factors leading to the emergence of zoonotic infectious diseases affecting humans, livestock, and wildlife (Morens et al. 2004; Jones et al. 2008). Understanding the dissemination of potential pathogens and role of specific hosts in the dissemination process is crucial if control measures are to be implemented to mitigate introduction of new infections into new environments and hosts. Indeed, emerging pathogens that originate from invasive species have caused numerous significant epidemics, including plague (*Yersinia pestis,* a bacterium) through rat introductions (Gage and Kosoy 2005), chytridiomycosis (*Batrachochytrium dendrobatidis*, a fungus) through amphibian introductions (Farrer et al. 2011), West Nile encephalitis (a virus) through bird or mosquito introduction, and avian malaria (*Plasmodium relictum*, a plasmodium) through mosquito introductions (Lapointe et al. 2012). New hosts can become pest species themselves, but when they invade a location they can carry with them pathogens that may evolve and adapt to novel hosts and locations if infection spillover occurs.

The harmful impact of invasive animal species on both natural and anthropogenic ecosystems has been well recognized for a long time (Zhang et al. 2006). One of the definitive signs of an invasive species is the niche expansion by introduction of the animals to areas where these species were absent in the past. During the last century some rat distributions have dramatically expanded, and the process of rats invading cities in different countries has created significant challenges (Khlyap et al. 2012). The results from a recent investigation conducted by Aplin and his collaborators suggested a strong phylogeographic pattern with well-differentiated lineages of rats native to South Asia, southern Indochina, and northern Indochina to East Asia (Aplin et al. 2011). One of the important conclusions from the study of the genetic structure of black rats conducted by Aplin et al. (2011) is the evidence suggesting that commensalism arose multiple times and in different populations of black rats. The establishment of rats in urban areas and their invasion into new territories can have significant implications for human health.

*Bartonella* bacteria are globally dispersed and are the causal agents of a wide variety of human and animal diseases (Anderson and Neuman 1997; Breitschwerdt and Kordick 2000; Boulouis et al. 2005; Kosoy et al. 2012). Bartonellae exhibit a unique parasitic strategy as hemotropic bacteria (Dehio et al. 2001; Birtles 2005; Kaiser et al. 2010) and have an extremely high prevalence and genetic diversity in certain mammalian orders, especially among rodent and bat species globally (Birtles et al. 1994; Kosoy et al. 1997; Ying et al. 2002; Castle et al. 2004; Jardine et al. 2005; Bai et al. 2011; Kosoy et al. 2012). Bartonella infection in rodents, and probably in bats, is characterized by slight if any clinical manifestations compared to the high prevalence of the infection (Bai and Kosoy 2012).

The geographical and phylogenetic relatedness of *Bartonella* species suggest that strain diversification is due to introductions into new locations with or without concomitant host shifts (Inoue et al. 2009; Berglund et al. 2010a,b; Paziewska et al. 2010). We recently argued for clarification of phylogeny of *Bartonella* species, in particular using the “complex sensu lato” approach for related *Bartonella* strains (Kosoy et al. 2012). *Bartonella elizabethae* complex sensu lato (CSL) is an assemblage of genotypes and strains associated with Old World rat genera *Rattus* and *Bandicota* (Heller et al. 1998; Ellis et al. 1999; Ying et al. 2002; Castle et al. 2004; Gundi et al. 2004, 2009; Kosoy et al. 2012). This group of *Bartonella* is hypothesized to have originated in the Old World, specifically in Southeast Asia, and emerged from Asia with two species of *Rattus* rats due to human activity, before becoming common and widespread in urban and rural environments around the world (Childs et al. 1999; Ellis et al. 1999). *Bartonella elizabethae*-CSL includes the majority of bartonella isolates from *Rattus* rats, including four recognized *Bartonella* species (*B. elizabethae, B. tribocorum, B. rattimassiliensis,* and *B. queenslandensis*) (Kosoy et al. 2012), with more than 20 *glt*A genotypes detected in Thailand (Bai et al. 2009) and a high diversity of *B. elizabethae*-CSL discovered in southern China (Ying et al. 2002). Strains of *Bartonella elizabethae*-CSL identified in high prevalence among *Bandicota bengalensis* and *R. rattus* rats in Bangladesh have had identical sequences to those from *Rattus* rats in Europe and the United States (Bai et al. 2007a,b). Later, one of these strains was demonstrated to be prevalent in commensal rats in Tel-Aviv, Israel (Harrus et al. 2009). Analysis of the isolates obtained from *Rattus* and *Bandicota* rats sampled from Bangladesh, Indonesia, Thailand, Vietnam, and China indicated that most *Bartonella* strains from rats belonged to *B. elizabethae*-CSL (Kosoy et al. 2012). *Bartonella* isolates from *R. norvegicus* from the USA and Peru and from *R. rattus* from Europe also belonged to *B. elizabethae*-CSL. Recent investigation of *Bartonella* species in rats from downtown Los Angeles, USA, demonstrated the similarity of most of them to strains prevalent in rats from Thailand (Gundi et al. 2012).

In this study we systematically test the hypotheses that (1) *B. elizabethae*-CSL evolution occurred in Asia, and (2) commensal rats play an important role in the dispersal of *B. elizabethae*-CSL bacteria around the globe, due to increased globalization and the dissemination of rats globally. For this aim, we use phylogenetic analyses to reconstruct the relationships between the bacteria and their geographic locations and hosts to infer the origins of bacteria.

## Material and Methods

The citrate synthase gene, *glt*A, is an especially popular and widely used molecular target for distinguishing between closely related *Bartonella* species and genotypes. Most laboratories working with *Bartonella* bacteria have successfully used this genetic marker, and consequently the majority of *Bartonella* sequences submitted to GenBank are derived from a 338 bp fragment of this gene. In addition to using sequences from GenBank, we were able to analyze sequences from the large database of the CDC Bartonella Laboratory (Fort Collins, CO), many of which have not been published. Therefore, we used 131 *glt*A sequences of *B. elizabethae*-CSL (Table S1) from 15 *Rattus* or *Bandicota* rat species (“true rats”) from 17 countries (Fig. S1), and 191 *glt*A sequences, including the previous 131 from true rats, of *B. elizabethae*-CSL (Table S1) from 27 rodent species from the Murinae subfamily from 17 countries in our analyses.

Sequences were aligned using ClustalX2.1 (Larkin et al. 2007). Sequence analysis was undertaken using Bayesian Markov chain Monte Carlo (MCMC) phylogenetic analysis using BEAST software (version 1.6.1) (Drummond and Rambaut 2007). Due to the nucleotide sequences being from coding regions, we used the SRD06 model; (Shapiro et al. 2006). The SRD06 model uses the Hasegawa, Kishino, and Tano (HKY) model of substitution with gamma (Γ)-distributed variation in rates among sites. Base frequencies were estimated and because of the low diversity in the *glt*A gene we estimated the nodes of the tree using substitutions per site. We assumed a constant population size and used uninformative priors throughout the analyses. A 120 × 10^6^ MCMC chain length was selected in BEAUti (version 1.6.1) and run in BEAST (version 1.6.1). Parameters were logged every 12000 trees. Models were accepted if parameter effective sample sizes (ESS) were >200 (but see Results and Discussion) and MCMC chains for parameter estimates had converged (Tracer, version 1.5). As per standard practice in MCMC phylogenetic analysis, the first 10% of maximum clade credibility (MCC) trees were discarded in TreeAnnotator (version 1.6.1) as burn-in before the chain has converged. Resulting MCC trees were visualized in FigTree (version 1.3.1). Sequence traits were categorized by state variables, and we used subregions within Eurasia (Southeast Asia, East Asia, South Asia, Western Asia, and Western Europe) and Africa (East, Central, and West), but did not subcategorize Australia and North or South America. We tested the hypothesis that these bacteria are derived from Asia and dispersed globally from there. We performed this analysis on two data sets, the first including *B. elizabethae*-CSL sequences derived only from true rats (*Rattus* and *Bandicota* genera), and the second from the wider Murinae subfamily. We also categorized the sequence traits based on the host from which they had been isolated (Table S1), in order to test the hypothesis that commensal rats, *R. rattus* and *R. norvegicus*, were involved in the ongoing transmission of *B. elizabethae*-CSL throughout the globe.

## Results

**Figure 1:**
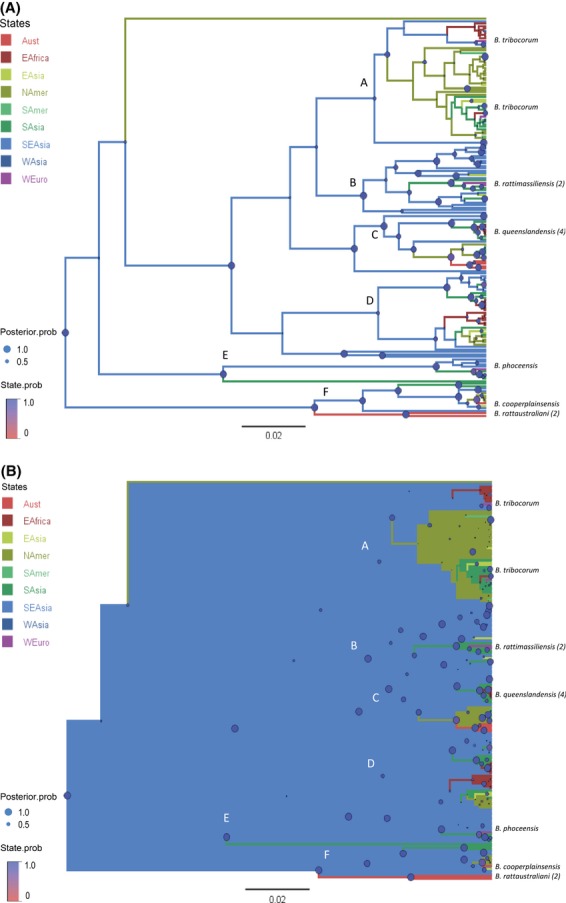
(A) Phylogeographic origin of 131 *Bartonella elizabethae* complex sensu lato *gltA* genome sequences from true rats analyzed. Posterior probabilities are shown as circles (•), scaled from 0 to 1 (posterior.prob), colored by the probability of the geographic origin (state.prob), with geographic region (states) shown by colored branches. Internal branches are colored by the region (states) with the most support (state.prob). The scale bar shows substitutions per site. Major clades discussed in the text are labeled A–F. The locations of previously named *Bartonella* are given: *Bartonella rattaustraliani; Bartonella queenslandensis; Bartonella coopersplainsensis; Bartonella tribocorum; Bartonella rattimassiliensis;* and *Bartonella phoceensis*. (B) As A, but with the background colored according to the geographic origin (states) with the most support (state.prob). Full details of the sequences used are given in Figure S2 and Table S1.

Our analyses of *B. elizabethae*-CSL tree topology and distance by substitutions per site in a Bayesian framework were successful, with good convergence and ESS for all parameter estimates (>200) for our analysis of *B. elizabethae*-CSL from true rats. Our analyses found a high degree of support for the Southeast Asian origins in *Bartonella* from *B. elizabethae*-CSL (Fig. 1, posterior probability [PP] = 1, state probability [SP] = 0.99). Furthermore, we found good support for the diversification of this complex within Southeast Asia before transmission events to numerous other locations globally (Fig. 1). Specifically, our analysis suggests that there have been numerous dispersal events within Asia and introductions from Asia to other regions, with six major clades with Southeast Asian isolates that appear to have been dispersed globally. Of note, clade A (Fig. 1) has a global distribution; with isolates detected in all regions we had data for, except Central Africa. Clade B is limited to Asia and Western Europe, whereas clade C has been dispersed to Africa, the Americas, and Australia. Outside Asia, clade D has been dispersed to Africa and North America. Clade E has limited geographic spread, with only a Eurasian distribution. Lastly, clade F appears to have a pan-Pacific distribution, with isolates detected in East Asia, Australia, and western North America. When we add the *B. elizabethae*-CSL sequences isolated from the other Murinae species, these clades remain intact (Figs. S3, S4) and Southeast Asia remains the most supported region of origin (PP = 1, SP = 0.99). The dispersal of those *B. elizabethae*-CSL sequences from other Murinae suggests that spillover from true rats to other Murinae genera (see host analysis below) has occurred in every region for which we have data. Including these sequences, however, reduced the ESS for the tree root height and the tree likelihood for codon position three to less than 100. We include these results, however, with this caveat in mind because all other parameter ESS were >200, in particular the overall tree likelihood ESS was 2721, the coalescent ESS 1595, and the region tree likelihood ESS 531.

**Figure 2:**
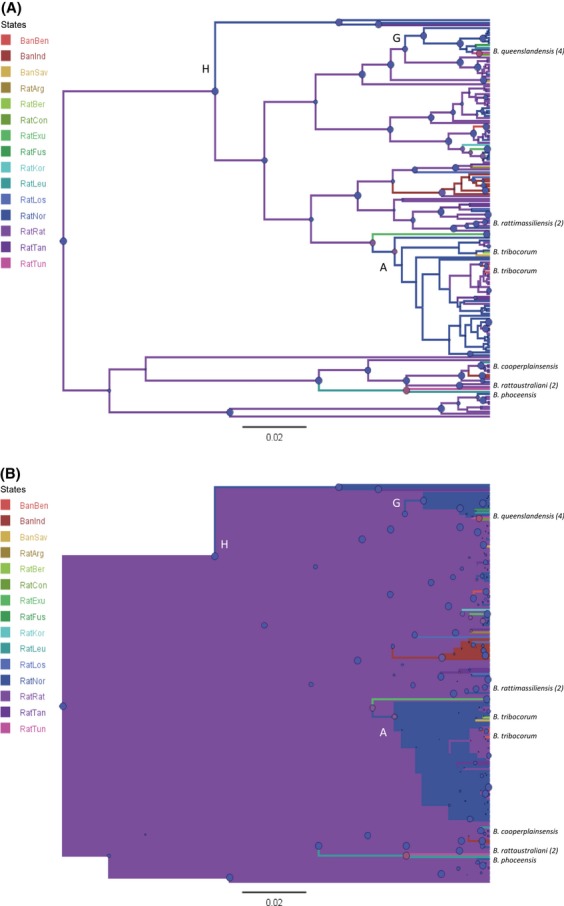
(A) Host genus origin of 131 *Bartonella elizabethae* complex sensu lato *gltA* genome sequences from true rats analyzed. Posterior probabilities are shown as circles (•), scaled from 0 to 1 (posterior.prob), colored by the probability of the host origin (state.prob), with host species (states) shown by colored branches. Internal branches are colored by the host (states) with the most support (state.prob). The scale bar shows substitutions per site. See Table S1 for full species names. Major clades discussed in the text are labeled A, G, and H. The locations of previously named *Bartonella* are given: *Bartonella rattaustraliani; Bartonella queenslandensis; Bartonella coopersplainsensis; Bartonella tribocorum; Bartonella rattimassiliensis;* and *Bartonella phoceensis*. (B) As A, but with the background colored according to the host origin (states) with the most support (state.prob). Full details of the sequences used are given in Figure S5 and Table S1.

Our phylogenetic analysis of the *B. elizabethae*-CSL sequence data estimating divergence by substitutions per site using host as a state variable also confirmed that commensal rats play a crucial role in the dispersal of *B. elizabethae*-CSL globally (Fig. 2). There was good convergence and ESS for all parameter estimates (>200) and high support for all states with *R. rattus* appearing as a host for the dispersal of *B. elizabethae*-CSL (Fig. 2, PP = 1, SP = 0.99). Indeed, *R. rattus*–derived *B. elizabethae*-CSL were basal for almost all clades and for all clades that appear to have been dispersed to other geographic locations and hosts (Fig. 2). However, *R. norvegicus* appears to play a major role in the dispersal of three clades (Fig. 2, clades A, G, H). The inclusion of the remaining sequences from other Murinae led to the same changes in ESS as for the region analysis, but further supported the hypothesis that dispersal of *B. elizabethae*-CSL by true rats leads to subsequent infection and evolution within other Murinae genera outside Southeast Asia, with multiple smaller clades or single sequences appearing throughout the tree (Figs. S6, S7).

## Discussion

We tested the hypothesis that early evolution and diversification of *B. elizabethae*-CSL bacteria took place in Southeast Asia, and have demonstrated that this is highly probable. We demonstrated that several dispersal events have introduced *B. elizabethae*-CSL bacteria into other parts of Asia, Europe, Africa, Australia, and the Americas multiple times. For example, clade A (Fig. 1) has been detected in rodents across the globe and was detected in all regions for which we had samples, except Central Africa. Our analysis shows commensal rats (Fig. 2), and in particular *R. rattus,* play an important role in the dispersal of *B. elizabethae*-CSL throughout the world. The black rat, *R. rattus*, has dispersed throughout the globe with major population expansions in prehistoric times and evidence for several commensalism events (Aplin et al. 2011). It is likely, therefore, that this species has played a role in the global dissemination of *Bartonella*, along with other bacterial infections such as plague (Gage and Kosoy 2005). The brown rat, *R. norvegicus*, however, is now more widely distributed and interestingly appears to have played a more important role in the dispersal of the most widely distributed clade, clade A, discovered in our analysis (Fig. 2).

Our analysis is particularly pertinent, given that many emerging diseases can be linked to the introduction of vectors or reservoir hosts to a new region. The number of introduction events suggested by our analysis and the importance of *R. norvegicus* in dispersing clade A suggest that this process is dynamic and ongoing. Our results, however, require numerous follow-up studies to strengthen our conclusions and understand the process of cross-species transmission and evolution within new hosts. In particular, substantial further sampling of different rodents globally is required. We have obtained sequences from 18 percent (12 of 66) *Rattus* species, and have limited our analysis to *B. elizabethae*-CSL, however, our sample represents just 1 percent (27 of ∼2277) murine species, and only 9 percent (17 of ∼190) countries. Therefore, substantially greater sampling should be undertaken to test our findings and determine the extent to which bartonellae are being disseminated by rodents, and the amount of cross-species transmission that is occurring. Similarly, commensal rodents are likely to be subject to biased sampling and therefore our results may be confounded by this. Furthermore, we used available sequence data, using partial *glt*A sequence alone. Using the year of isolation in our phylogenetic analysis, we were unable to get reliable estimates of mutation rates for *B. elizabethae*-CSL and were unable to estimate divergence times with any confidence (data not shown). Future analyses should aim to build upon our studies, by incorporating multilocus sequence typing (MLST) and/or full-genome sequences into the phylogenetic analyses as more become available. These analyses may be able to better estimate divergence times, and thus determine if the divergence dates for specific clades match with human exploration and colonization of various global locations, such as the colonization of the Americas by Europeans.

Full MLST and genome analyses will also ensure that results are not confounded by lateral gene transfers (LGT) and recombination events. *Bartonella grahamii* has high recombination rates and evidence for LGT, showing genetic exchanges occurring among different strains or species that share hosts in rodent communities (Berglund et al. 2009, 2010b). LGT has been inferred among *B. grahamii* strains and between *B. grahamii* and *B. taylorii*, another rodent-borne species that frequently infects the same hosts as *B. grahamii* (Paziewska et al. 2010). However, analyses of *B. grahamii* strain genomic data suggest that geographic diversification of rodent-borne bartonellae is due to genes located within a region of the genome containing phage sequences that experience runoff replication and thus potentially diversification and adaptation to new ecological niches within hosts (Berglund et al. 2009). Analyses of *B. grahamii* isolate genomes from just three locations within 30 km of one another showed diversification, although the strains had low sequence diversity on the whole (Berglund et al. 2010a). Therefore, use of genome regions such as these may be informative.

Generally, however, we believe our results are important because they demonstrate the role that human-mediated dissemination of invasive hosts can play on pathogen–host relationship. Our analyses suggest that dissemination of *B. elizabethae*-CSL may have been widespread throughout Asia and from Asia to Africa, Australia, Europe, and the Americas. Phylogenetic analysis of *B. elizabethae*-CSL supports the hypothesis of coadaptation among at least some groups, such as rat-associated strains, but *Bartonella*–rodent host associations suggest that cospeciation varies among studies and around the world. Prior to our study an association between *B. elizabethae*-CSL bacteria in *Rattus* rats in southern China (Ying et al. 2002) and *Bandicota* rats in Thailand (Castle et al. 2004) has been discovered; however, in Europe a single species of *Bartonella* commonly infects multiple rodent species (Birtles et al. 2001; Holmberg et al. 2003) and spillover has been reported commonly (Bai et al. 2007a,b). *Bartonella grahamii* and associated species are globally distributed and commonly isolated from a very broad range of rodent genera, including *Apodemus, Mus,* and *Rattus* spp. (Bajer et al. 2001; Inoue et al. 2009). Inoue et al. (2009) hypothesize that *B. grahamii* originated in North America and isolates were distributed to European countries with adaptation to various rodent hosts. Thus, the dispersal of hosts may be leading to increased parasitism of rodent hosts throughout the world. Typically, human translocated invasive species are adaptable species, such as *R. rattus* and *R. norvegicus,* that can have devastating consequences on flora and fauna by themselves. However, they can also introduce their pathogens, which may lead to altered parasitism of local species and introduction of zoonotic diseases (which are not mutually exclusive). For example, *Bartonella* infection of field voles, *Microtus agrestis*, can lead to increased probability of infection with cowpox virus, but decreased probability of infection with *Anaplasma* and *Babesia* species (Telfer et al. 2010). Thus, introduction of parasites through novel hosts may lead to altered parasite community dynamics beyond those that appear most obvious, such as the introduction of a known zoonotic infection or pest host species. Therefore, we recommend that further sampling is necessary at local levels to elucidate how bacterial pathogens adapt to new hosts once cross-species transmission events have occurred.

In summary, we believe our analyses provide a useful framework for future analyses to test hypotheses regarding the importance of hosts in pathogen transmission through genotype sharing, and we found support for the evolution of *B. elizabethae*-CSL in Southeast Asia with likely transmission of this bacterial complex by commensal rodents throughout the globe. 

## Data Accessibility

All sequences have GenBank accession numbers (see Table S1).

## Supporting Information

**Figure S1.** Geographic origin of the 191 *Bartonella gltA* genome sequences used in this study.

**Figure S2.** Phylogeographic origin of the 131 *Bartonella elizabethae* complex sensu lato *gltA* genome sequences from true rats analyzed in a Bayesian framework, with full sequence details at the branch tips. Details are: Region_Species_CountryGenBankReference_YearIsolated. For full details, see table S1. All other features match those in Figure 1. Posterior probabilities are shown as circles (•), scaled from 0 to 1 (posterior.prob), colored by the probability of the geographic origin (state.prob), with geographic region (states) shown by colored branches. Internal branches are colored by the region (states) with the most support (state.prob). The scale bar shows substitutions per site.

**Figure S3.** (A) Phylogeographic origin of 191 *Bartonella elizabethae* complex sensu lato *gltA* genome sequences from Murinae analyzed. Posterior probabilities are shown as circles (•), scaled from 0 to 1 (posterior.prob), colored by the probability of the geographic origin (state.prob), with geographic region (states) shown by colored branches. Internal branches are colored by the region (states) with the most support (state.prob). The scale bar shows substitutions per site. Major clades discussed in the text are labeled A-F. The location of previously named *Bartonella* is given: *Bartonella rattaustraliani; Bartonella queenslandensis; Bartonella coopersplainsensis; Bartonella tribocorum; Bartonella rattimassiliensis;* and *Bartonella phoceensis*. (B) As A, but with the background colored according to the geographic origin (states) with most support (state.prob). Full details of the sequences used are given in Figure S4 and Table S1.

**Figure S4.** Phylogeographic origin of the 191 *Bartonella elizabethae* complex sensu lato *gltA* genome sequences from Murinae analyzed in a Bayesian framework, with full sequence details at the branch tips. Details are: Region_Species_CountryGenBankReference_YearIsolated. For full details, see table S1. All other features match those in Figure S3. Posterior probabilities are shown as circles (•), scaled from 0 to 1 (posterior.prob), colored by the probability of the geographic origin (state.prob), with geographic region (states) shown by colored branches. Internal branches are colored by the region (states) with the most support (state.prob). The scale bar shows substitutions per site.

**Figure S5.** Host origin of the 131 *Bartonella elizabethae* complex sensu lato *gltA* genome sequences from true rats analyzed in a Bayesian framework, with full sequence details at the branch tips. Details are: Region_Species_CountryGenBankReference_YearIsolated. For full details, see table S1. All other features match those in Figure 2. Posterior probabilities are shown as circles (•), scaled from 0 to 1 (posterior.prob), colored by the probability of the host origin (state.prob), with host (states) shown by colored branches. Internal branches are colored by the host (states) with the most support (state.prob). The scale bar shows substitutions per site.

**Figure S6.** (A) Host genus origin of 191 *Bartonella elizabethae* complex sensu lato *gltA* genome sequences from Murinae analyzed. Posterior probabilities are shown as circles (•), scaled from 0 to 1 (posterior.prob), colored by the probability of the host origin (state.prob), with host (states) shown by colored branches. Internal branches are colored by the host (states) with most support (state.prob). The scale bar shows substitutions per site. Major clades discussed in the text are labeled A, G and H. The location of previously named *Bartonella* is given: *Bartonella rattaustraliani; Bartonella queenslandensis; Bartonella coopersplainsensis; Bartonella tribocorum; Bartonella rattimassiliensis;* and *Bartonella phoceensis*. B) As above, but with the background colored according to the host origin (states) with the most support (state.prob). Full details of the sequences used are given in Figure S7 and Table S1.

**Figure S7.** Host genus origin of the 191 *Bartonella elizabethae* complex sensu late *gltA* genome sequences from Murinae analyzed in a Bayesian framework, with full sequence details at the branch tips. Details are: Region_Species_CountryGenBankReference_YearIsolated. All other features match those in Figure S6. Posterior probabilities are shown as circles (•), scaled from 0 to 1 (posterior.prob), colored by the probability of the host origin (state.prob), with host (states) shown by colored branches. Internal branches are colored by the host (states) with the most support (state.prob). The scale bar shows substitutions per site.

**Table S1.** The *Bartonella elizabethae* complex sensu lato citrate synthase gene, *glt*A, sequence data used in this analysis.
