# Supplementary figures and images for: Evolutionary history of rat-borne Bartonella: the importance of commensal rats in the dissemination of bacterial infections globally
Source: Ecol Evol. 2013 Aug 6;3(10):3195–203. doi: 10.1002/ece3.702 (PMC3797470; doi:10.1002/ece3.702)

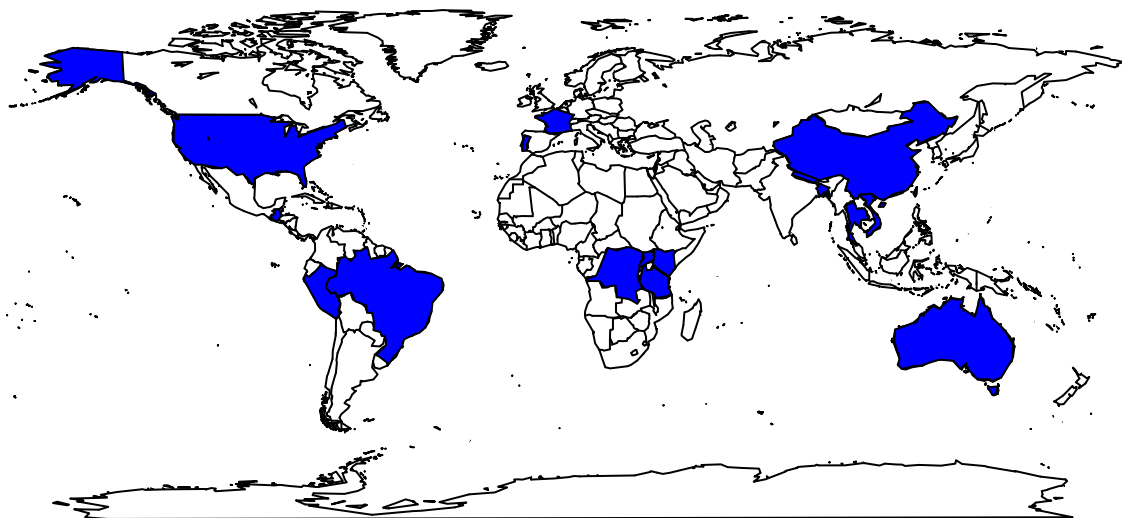

Supplement: Supplementary file 1 [file ece30003-3195-SD1.pdf]

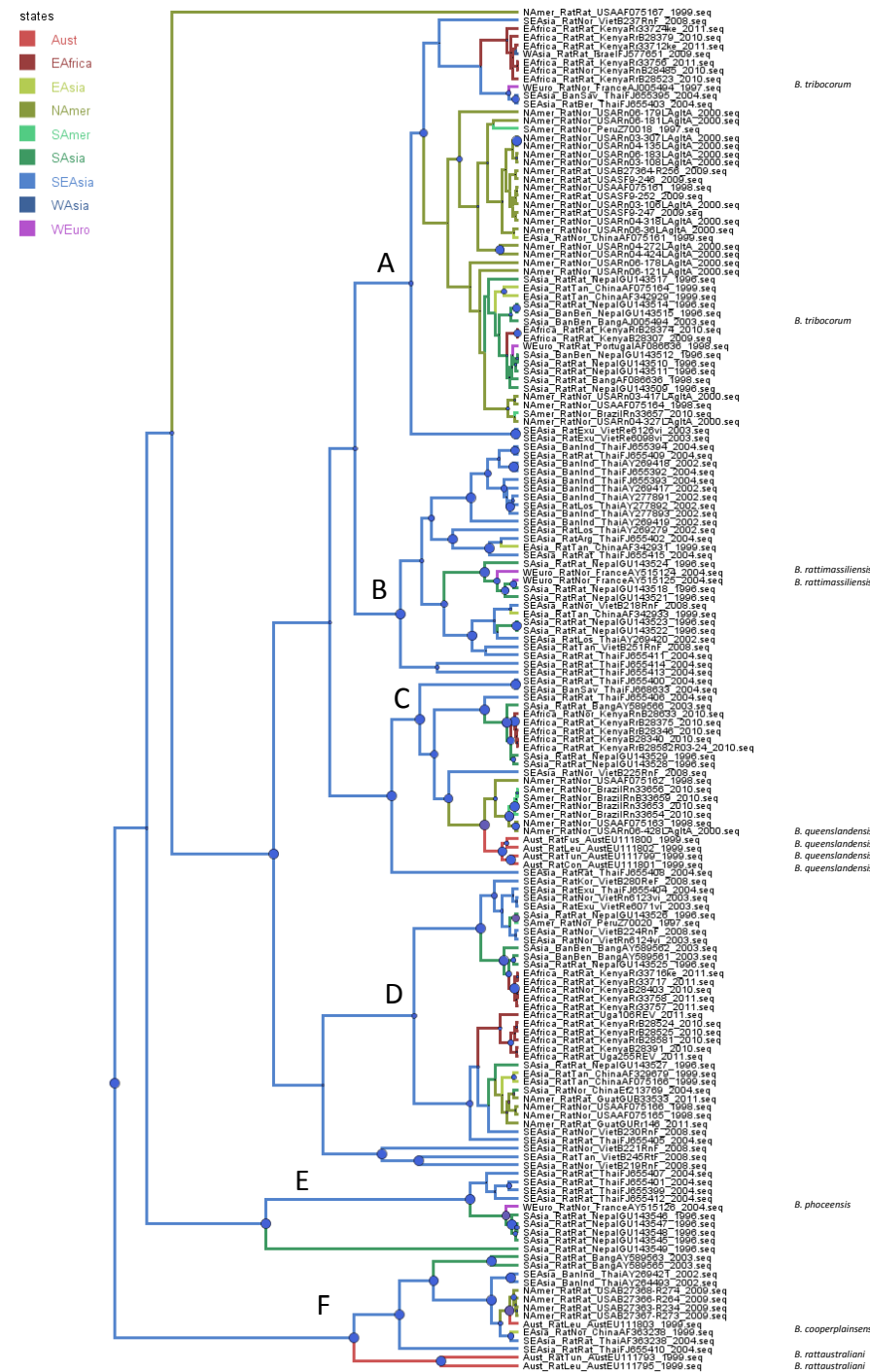

Supplement: Supplementary file 2 [file ece30003-3195-SD2.pdf]

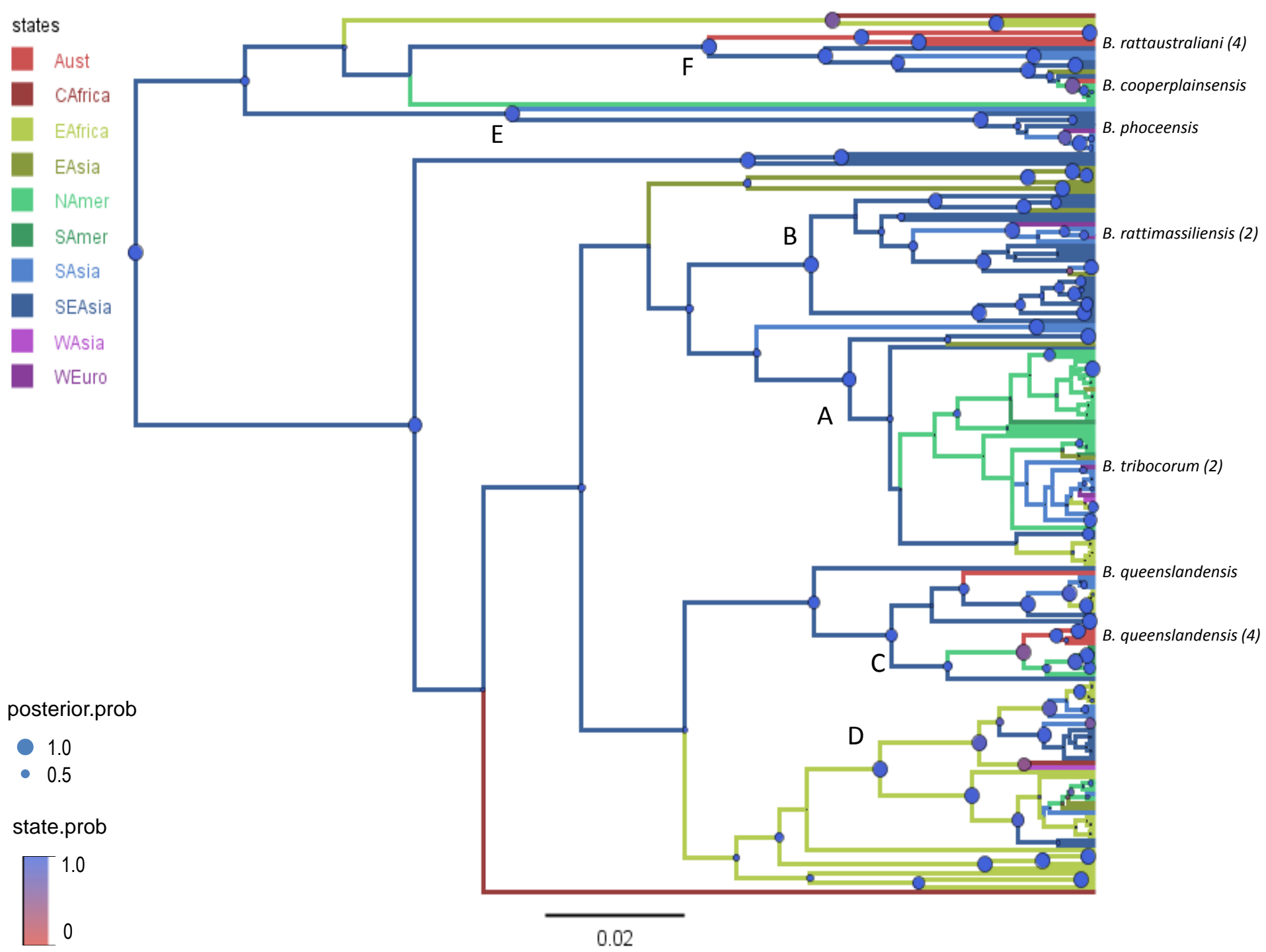

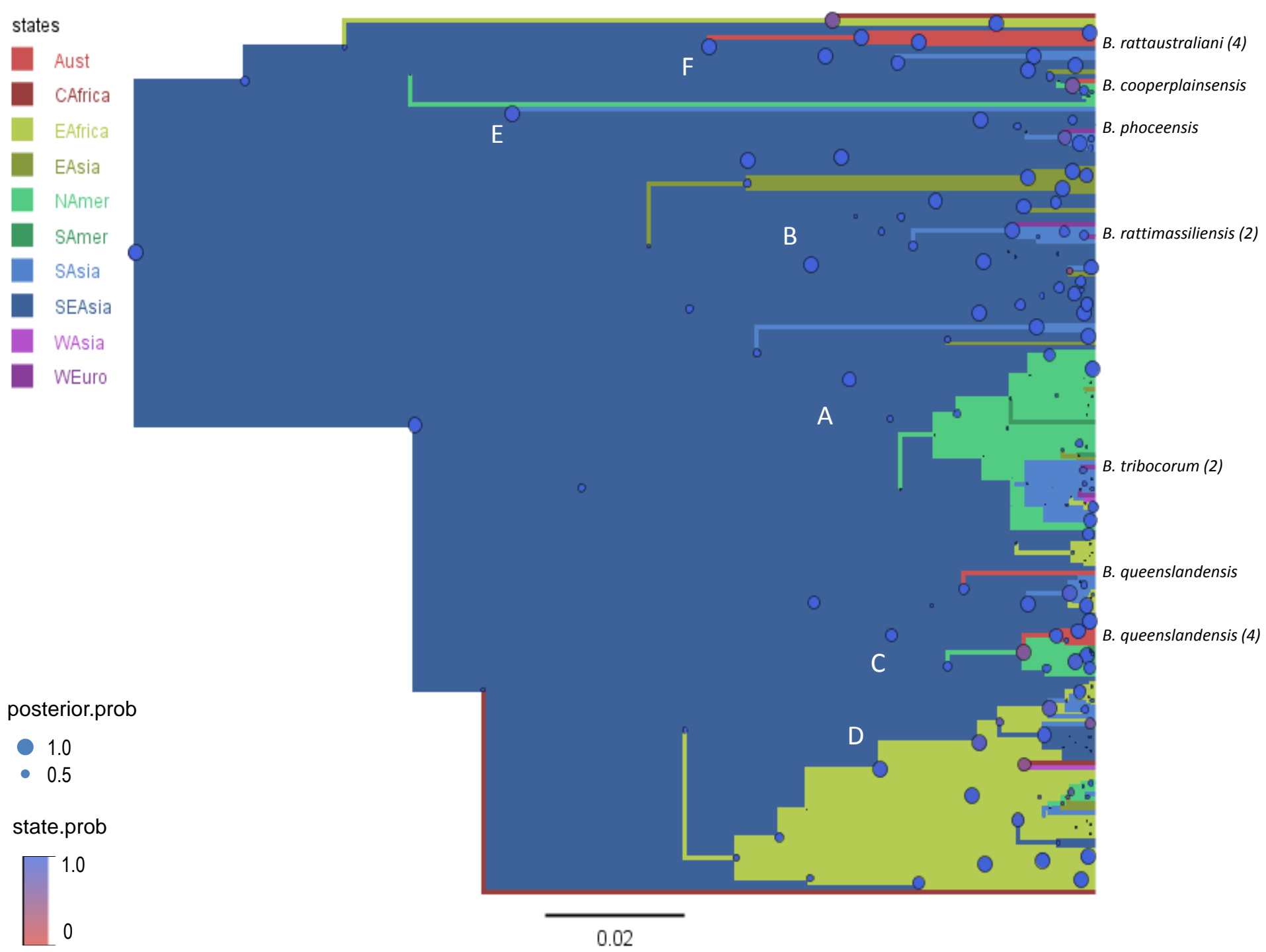

Supplement: Supplementary file 3 [file ece30003-3195-SD3.pdf]

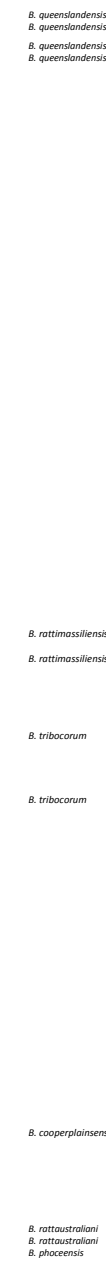

Supplement: Supplementary file 5 [file ece30003-3195-SD5.pdf]

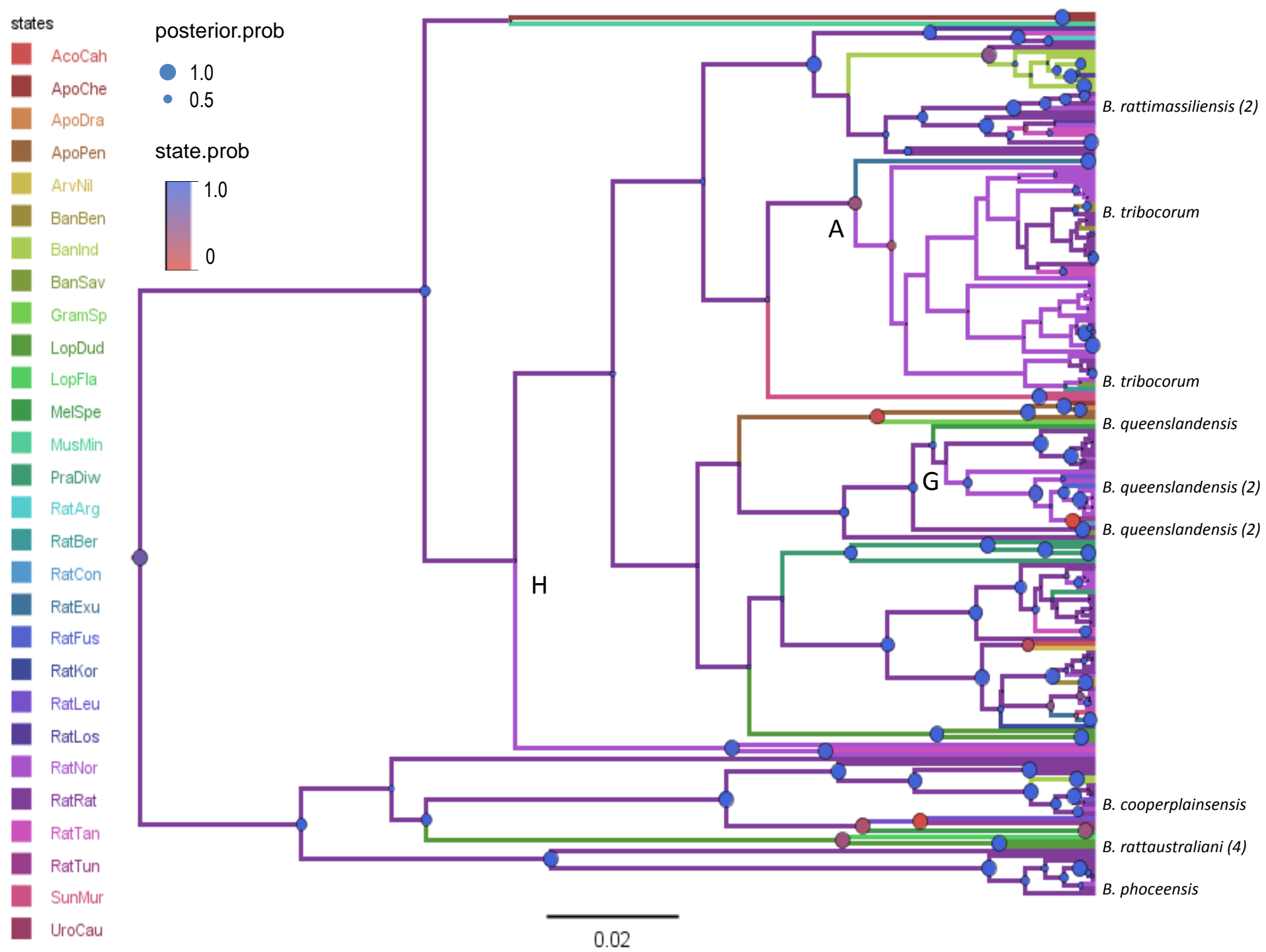

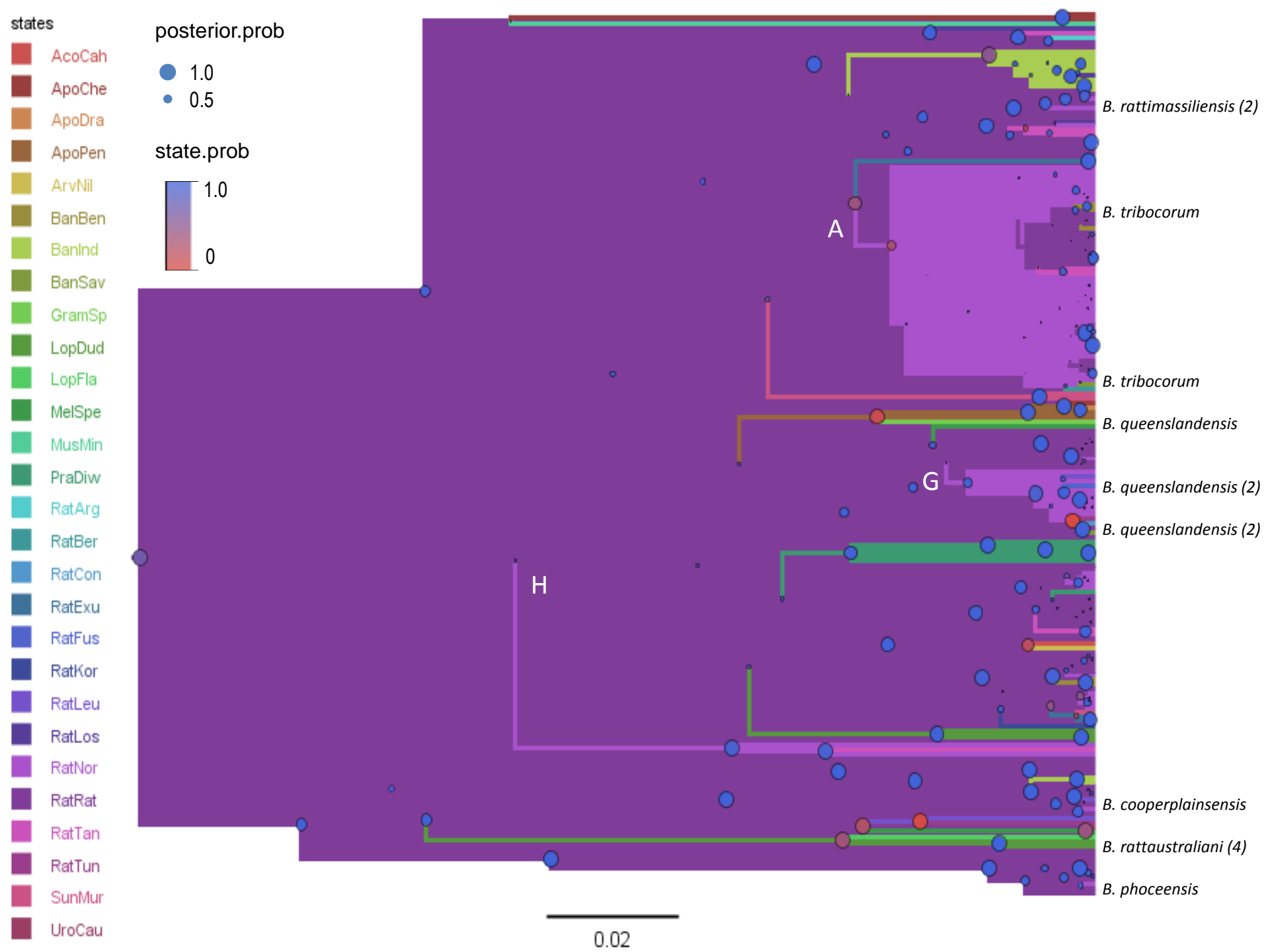

Supplement: Supplementary file 6 [file ece30003-3195-SD6.pdf]
