# Supplementary material for: Evolutionary history of rat-borne Bartonella: the importance of commensal rats in the dissemination of bacterial infections globally
Source: Ecol Evol. 2013 Aug 6;3(10):3195–203. doi: 10.1002/ece3.702 (PMC3797470; doi:10.1002/ece3.702)
Supplement: Supplementary file 4 [file ece30003-3195-SD4.pdf]

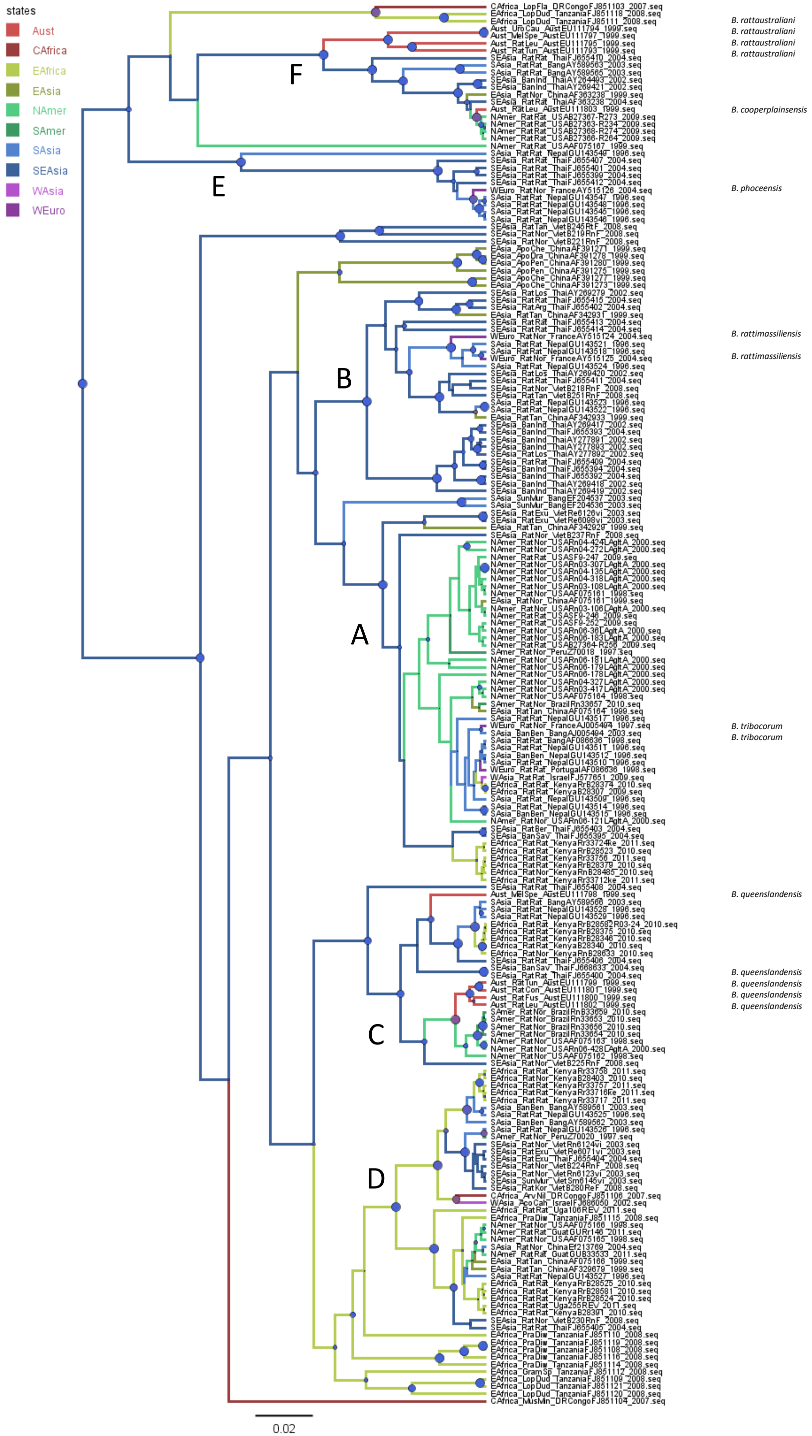
states
Aust
CAfrica
EAfrica
EAsia
NAmer
SAmer
SAsia
SEAsia
WAsia
WEuro
F
E
B
A
C
D
B. rattaustraliani
B. rattaustraliani
B. rattaustraliani
B. rattaustraliani
B. cooperplainsensis
B. phoceensis
B. rattimassiliensis
B. rattimassiliensis
B. tribocorum
B. tribocorum
B. queenslandensis
B. queenslandensis
B. queenslandensis
B. queenslandensis
B. queenslandensis
0.02
