# Supplementary material for: Evolutionary history of rat-borne Bartonella: the importance of commensal rats in the dissemination of bacterial infections globally
Source: Ecol Evol. 2013 Aug 6;3(10):3195–203. doi: 10.1002/ece3.702 (PMC3797470; doi:10.1002/ece3.702)
Supplement: Supplementary file 7 [file ece30003-3195-SD7.pdf]

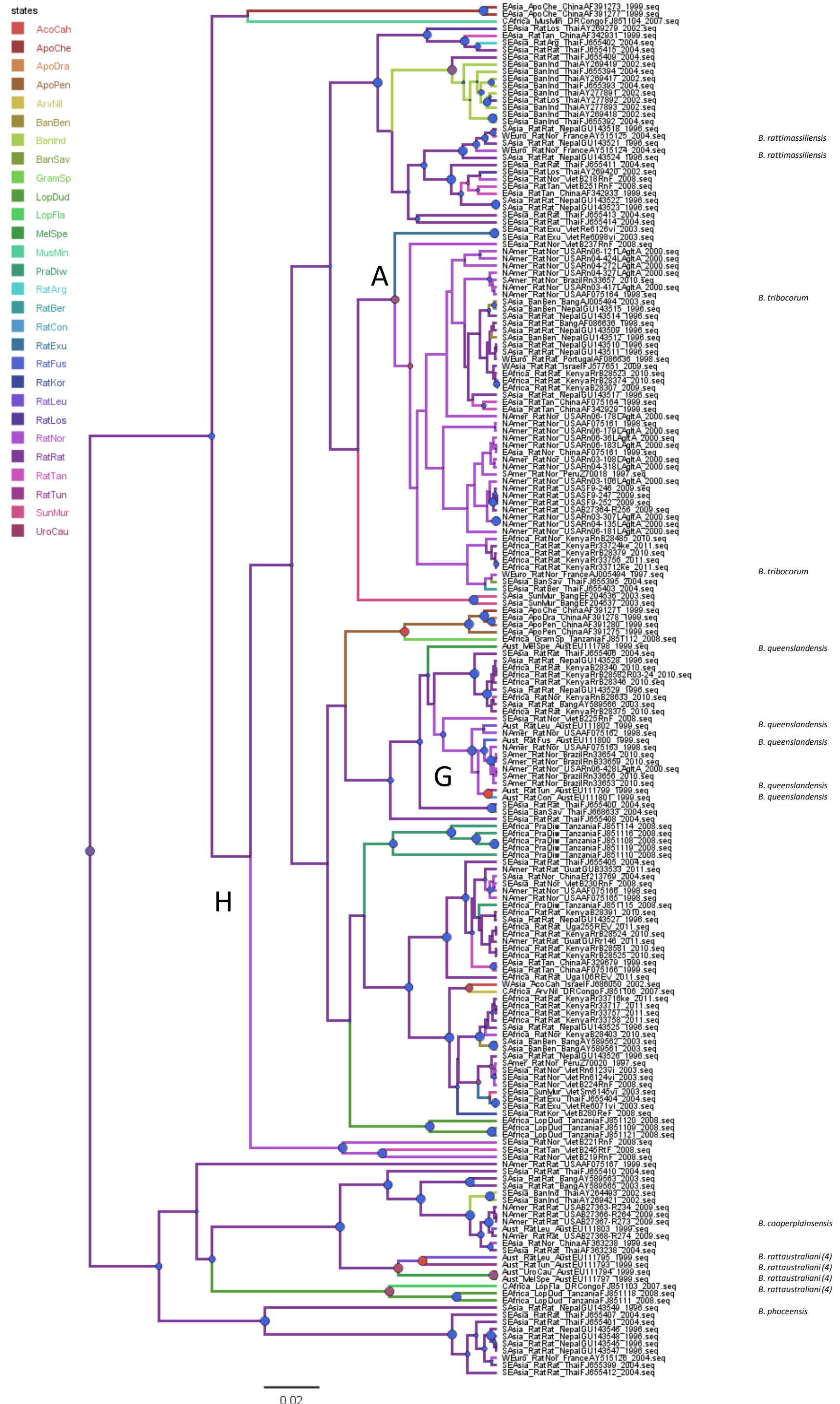
states
AcoCah
ApoChe
ApoDra
ApoPen
ArvNil
BanBen
BanInd
BanSav
GramSp
LopDud
LopFla
MelSpe
MusMin
PraDiw
RatArg
RatBer
RatCon
RatExu
RatFus
RatKor
RatLeu
RatLos
RatNor
RatRat
RatTan
RatTun
SunMur
UroCau
A
G
H
B. rattimassiliensis
B. rattimassiliensis
B. tribocorum
B. tribocorum
B. queenslandensis
B. queenslandensis
B. queenslandensis
B. queenslandensis
B. queenslandensis
B. cooperplainsensis
B. rattaustraliani (4)
B. rattaustraliani (4)
B. rattaustraliani (4)
B. rattaustraliani (4)
B. phoceensis
0.02
