# Supplementary material for: Evolutionary history of rat-borne Bartonella: the importance of commensal rats in the dissemination of bacterial infections globally
Source: Ecol Evol. 2013 Aug 6;3(10):3195–203. doi: 10.1002/ece3.702 (PMC3797470; doi:10.1002/ece3.702)
Supplement: Supplementary file 14 [file ece30003-3195-SD14.docx]

**Supplementary information**

**Figure legends**

Figure S1. Geographic origin of the 191 *Bartonella* *gltA* genome sequences used in this study

Figure S2. Phylogeographic origin of the 131 *Bartonella elizabethae* complex sensu lato *gltA* genome sequences from true rats analyzed in a Bayesian framework, with full sequence details at the branch tips. Details are: Region_Species_CountryGenBankReference_YearIsolated. For full details, see table S1. All other features match those in Figure 1. Posterior probabilities are shown as circles (●), scaled from 0 to 1 (posterior.prob), colored by the probability of the geographic origin (state.prob), with geographic region (states) shown by colored branches. Internal branches are colored by the region (states) with the most support (state.prob). The scale bar shows substitutions per site.

Figure S3. A) Phylogeographic origin of 191 *Bartonella elizabethae* complex sensu lato *gltA* genome sequences from Murinae analyzed. Posterior probabilities are shown as circles (●), scaled from 0 to 1 (posterior.prob), colored by the probability of the geographic origin (state.prob), with geographic region (states) shown by colored branches. Internal branches are colored by the region (states) with the most support (state.prob). The scale bar shows substitutions per site. Major clades discussed in the text are labeled A-F. The location of previously named *Bartonella* is given: *Bartonella rattaustraliani; Bartonella queenslandensis; Bartonella coopersplainsensis; Bartonella tribocorum; Bartonella rattimassiliensis;* and *Bartonella phoceensis*. B) As A, but with the background colored according to the geographic origin (states) with most support (state.prob). Full details of the sequences used are given in Figure S4 and Table S1.

Figure S4. Phylogeographic origin of the 191 *Bartonella elizabethae* complex sensu lato *gltA* genome sequences from Murinae analyzed in a Bayesian framework, with full sequence details at the branch tips. Details are: Region_Species_CountryGenBankReference_YearIsolated. For full details, see table S1. All other features match those in Figure S3. Posterior probabilities are shown as circles (●), scaled from 0 to 1 (posterior.prob), colored by the probability of the geographic origin (state.prob), with geographic region (states) shown by colored branches. Internal branches are colored by the region (states) with the most support (state.prob). The scale bar shows substitutions per site.

Figure S5. Host origin of the 131 *Bartonella elizabethae* complex sensu lato *gltA* genome sequences from true rats analyzed in a Bayesian framework, with full sequence details at the branch tips. Details are: Region_Species_CountryGenBankReference_YearIsolated. For full details, see table S1. All other features match those in Figure 2. Posterior probabilities are shown as circles (●), scaled from 0 to 1 (posterior.prob), colored by the probability of the host origin (state.prob), with host (states) shown by colored branches. Internal branches are colored by the host (states) with the most support (state.prob). The scale bar shows substitutions per site.

Figure S6. A) Host genus origin of 191 *Bartonella elizabethae* complex sensu lato *gltA* genome sequences from Murinae analyzed. Posterior probabilities are shown as circles (●), scaled from 0 to 1 (posterior.prob), colored by the probability of the host origin (state.prob), with host (states) shown by colored branches. Internal branches are colored by the host (states) with most support (state.prob). The scale bar shows substitutions per site. Major clades discussed in the text are labeled A, G and H. The location of previously named *Bartonella* is given: *Bartonella rattaustraliani; Bartonella queenslandensis; Bartonella coopersplainsensis; Bartonella tribocorum; Bartonella rattimassiliensis;* and *Bartonella phoceensis*. B) As above, but with the background colored according to the host origin (states) with the most support (state.prob). Full details of the sequences used are given in Figure S7 and Table S1.

Figure S7. Host genus origin of the 191 *Bartonella elizabethae* complex sensu late *gltA* genome sequences from Murinae analyzed in a Bayesian framework, with full sequence details at the branch tips. Details are: Region_Species_CountryGenBankReference_YearIsolated. All other features match those in Figure S6. Posterior probabilities are shown as circles (●), scaled from 0 to 1 (posterior.prob), colored by the probability of the host origin (state.prob), with host (states) shown by colored branches. Internal branches are colored by the host (states) with the most support (state.prob). The scale bar shows substitutions per site.

**Table legend**

Table S1. The *Bartonella elizabethae* complex sensu lato citrate synthase gene, *glt*A, sequence data used in this analysis.

| **Sequence Name** | **Host species** | **Country** | **Date collection** | **GenBank #** | **GenBank Organism name** |
| --- | --- | --- | --- | --- | --- |
| Rattaustraliani AUST/NH4 | *Rattus tunneyi* | Australia | 1999 | EU111793 | Bartonella rattaustraliani |
| Rattaustraliani AUST/NH9 | *Uromys caudimaculatus* | Australia | 1999 | EU111794 | Bartonella rattaustraliani |
| Rattaustraliani AUST/NH10 | *Rattus leucopus* | Australia | 1999 | EU111795 | Bartonella rattaustraliani |
| Rattaustraliani AUST/NH18 | *Melomys sp* | Australia | 1999 | EU111797 | Bartonella rattaustraliani |
| Queenslandensis AUST/NH5 | *Rattus tunneyi* | Australia | 1999 | EU111799 | Bartonella queenslandensis |
| Queenslandensis AUST/NH8 | *Rattus fuscipes* | Australia | 1999 | EU111800 | Bartonella queenslandensis |
| Queenslandensis AUST/NH11 | *Rattus conatus* | Australia | 1999 | EU111801 | Bartonella queenslandensis |
| Queenslandensis AUST/NH12 | *Melomys sp* | Australia | 1999 | EU111798 | Bartonella queenslandensis |
| Queenslandensis AUST/NH15 | *Rattus leucopus* | Australia | 1999 | EU111802 | Bartonella queenslandensis |
| Coopersplainensis AUST/NH20 | *Rattus leucopus* | Australia | 1999 | EU111803 | Bartonella coopersplainsensis |
| Bb6966bgl | *Bandicota bengalensis* | Bangladesh | 2003 | AY589561 | Bartonella spp. |
| Bb7700bgl | *Bandicota bengalensis* | Bangladesh | 2003 | AY589562 | Bartonella spp. |
| Rr7684bgl | *Rattus rattus* | Bangladesh | 2003 | AY589563 | Bartonella spp. |
| Rr7703bgl | *Rattus rattus* | Bangladesh | 2003 | AY589565 | Bartonella spp. |
| Rr7624bgl | *Rattus rattus* | Bangladesh | 2003 | AY589566 | Bartonella spp. |
| Rr7107bgl | *Rattus rattus* | Bangladesh | 2003 | AF086636 | Bartonella spp. |
| Bb7612bgl | *Bandicota bengalensis* | Bangladesh | 2003 | AJ005494 | Bartonella tribocorum |
| Sm7680bgl | *Suncus murinus* | Bangladesh | 2003 | EF204536 | Bartonella spp. |
| Sm7688bg | *Suncus murinus* | Bangladesh | 2003 | EF204537 | Bartonella spp. |
| Rn33653-brazil | *Rattus norvegicus* | Brazil | 2010 | KC763961 | Bartonella spp. |
| Rn33657-brazil | *Rattus norvegicus* | Brazil | 2010 | AF075164 | Bartonella spp. |
| Rn33654-brazil | *Rattus norvegicus* | Brazil | 2010 | KC763961 | Bartonella spp. |
| Rn33656-brazil | *Rattus norvegicus* | Brazil | 2010 | KC763961 | Bartonella spp. |
| RnB33659-brazil | *Rattus norvegicus* | Brazil | 2010 | KC763961 | Bartonella spp. |
| Ac1692yn | *Apodemus chevrieri* | China | 1999 | AF391271 | Bartonella spp. |
| Ac1825yn | *Apodemus chevrieri* | China | 1999 | AF391273 | Bartonella spp. |
| Ac1727yn | *Apodemus chevrieri* | China | 1999 | AF391277 | Bartonella spp. |
| Ad1734yn | *Apodemus draco* | China | 1999 | AF391278 | Bartonella spp. |
| Ap1707yn | *Apodemus peninsulae* | China | 1999 | AF391275 | Bartonella spp. |
| Ap1714yn | *Apodemus peninsulae* | China | 1999 | AF391280 | Bartonella spp. |
| Rf1449yn | *Rattus norvegicus* | China | 1999 | AF075161 | Bartonella spp. |
| Rf1554yn | *R. t. flavipectus* | China | 1999 | AF342931 | Bartonella spp. |
| Rf1557yn | *R. t. flavipectus* | China | 1999 | AF075164 | Bartonella spp. |
| Rf1561yn | *R. t. flavipectus* | China | 1999 | AF342933 | Bartonella spp. |
| Rf1563yn | *R. t. flavipectus* | China | 1999 | AF329679 | Bartonella spp. |
| Rf1570yn | *R. t. flavipectus* | China | 1999 | AF342929 | Bartonella spp. |
| Rf1575yn | *R. t. flavipectus* | China | 1999 | AF075166 | Bartonella spp. |
| Rn1691yn | *Rattus norvegicus* | China | 1999 | AF363238 | Bartonella spp. |
| Rn34bj | *Rattus norvegicus* | China | 2007 | EF213769 | Bartonella spp. |
| AnDRC615_CA24 | *Arvicanthis niloticus* | DRCongo | 2007 | FJ851106 | Bartonella spp. |
| LfDRC601_CA1 | *Lofhuromys flavopunctatus* | DRCongo | 2007 | FJ851103 | Bartonella spp. |
| MmDRC625_CA13 | *Mus minutoides* | DRCongo | 2007 | FJ851104 | Bartonella spp. |
| Tribocorum | *Rattus norvegicus* | France | 1997 | AJ005494 | Bartonella tribocorum |
| Rattimassiliensis 15908 | *Rattus norvegicus* | France | 2004 | AY515124 | Bartonella rattimassiliensis |
| Rattimassiliensis 16115 | *Rattus norvegicus* | France | 2004 | AY515125 | Bartonella rattimassiliensis |
| Phoceensis | *Rattus norvegicus* | France | 2004 | AY515126 | Bartonella phoceensis |
| Rattus GU B33533 | *Rattus rattus* | Guatemala | 2011 | EF213769 | Bartonella spp. |
| Rattus GU Rr146 | *Rattus rattus* | Guatemala | 2011 | AF075166 | Bartonella spp. |
| Tel Aviv strain | *Rattus rattus* | Israel | 2009 | FJ577651 | Bartonella spp. |
| A cahirinus | *Acomys cahirinus* | Israel | 2002 | FJ686050 | Bartonella spp. |
| Rr 33712ke | *Rattus rattus* | Kenya | 2011 | KC763963 | Bartonella spp. |
| Rr 33716ke | *Rattus rattus* | Kenya | 2011 | KC763964 | Bartonella spp. |
| Rr 33724ke | *Rattus rattus* | Kenya | 2011 | FJ577651 | Bartonella spp. |
| Rn B28403 RO2-11 | *Rattus norvegicus* | Kenya | 2010 | KC763966 | Bartonella spp. |
| Rr B28307 RO1-1 | *Rattus rattus* | Kenya | 2010 | AF086636 | Bartonella spp. |
| Rr B28340 RO1-21 | *Rattus rattus* | Kenya | 2010 | KC763967 | Bartonella spp. |
| Rr B28391 RO1-80 | *Rattus rattus* | Kenya | 2010 | KC763968 | Bartonella spp. |
| Rr B28582 RO3-24 | *Rattus rattus* | Kenya | 2010 | KC763962 | Bartonella spp. |
| RnB28485 | *Rattus norvegicus* | Kenya | 2010 | KC763969 | Bartonella spp. |
| RnB28633 | *Rattus norvegicus* | Kenya | 2010 | KC763962 | Bartonella spp. |
| Rr33717 | *Rattus rattus* | Kenya | 2011 | KC763964 | Bartonella spp. |
| Rr33756 | *Rattus rattus* | Kenya | 2011 | KC763963 | Bartonella spp. |
| Rr33757 | *Rattus rattus* | Kenya | 2011 | KC763966 | Bartonella spp. |
| Rr33758 | *Rattus rattus* | Kenya | 2011 | KC763966 | Bartonella spp. |
| RrB28346 R01-40 | *Rattus rattus* | Kenya | 2010 | KC763967 | Bartonella spp. |
| RrB28374 R01-68 | *Rattus rattus* | Kenya | 2010 | AF086636 | Bartonella spp. |
| RrB28375 R01-39 | *Rattus rattus* | Kenya | 2010 | KC763962 | Bartonella spp. |
| RrB28379 R01-66 | *Rattus rattus* | Kenya | 2010 | KC763969 | Bartonella spp. |
| RrB28523 R03-20 | *Rattus rattus* | Kenya | 2010 | KC763969 | Bartonella spp. |
| RrB28524 R03-21 | *Rattus rattus* | Kenya | 2010 | KC763968 | Bartonella spp. |
| RrB28525 R03-22 | *Rattus rattus* | Kenya | 2010 | KC763968 | Bartonella spp. |
| RrB28581 R02-66 | *Rattus rattus* | Kenya | 2010 | KC763968 | Bartonella spp. |
| Bandicota GU143512 | *Bandicota bengalensis* | Nepal | 1996 | GU143512 | Bartonella spp. |
| Bandicota GU143515 | *Bandicota bengalensis* | Nepal | 1996 | GU143515 | Bartonella spp. |
| Rattus GU143509 | *Rattus rattus brunnesculus* | Nepal | 1996 | GU143509 | Bartonella spp. |
| Rattus GU143510 | *Rattus rattus brunnesculus* | Nepal | 1996 | GU143510 | Bartonella spp. |
| Rattus GU143511 | *Rattus rattus brunnesculus* | Nepal | 1996 | GU143511 | Bartonella spp. |
| Rattus GU143514 | *Rattus rattus brunnesculus* | Nepal | 1996 | GU143514 | Bartonella spp. |
| Rattus GU143517 | *Rattus rattus brunnesculus* | Nepal | 1996 | GU143517 | Bartonella spp. |
| Rattus GU143518 | *Rattus rattus brunnesculus* | Nepal | 1996 | GU143518 | Bartonella spp. |
| Rattus GU143521 | *Rattus rattus brunnesculus* | Nepal | 1996 | GU143521 | Bartonella spp. |
| Rattus GU143522 | *Rattus rattus brunnesculus* | Nepal | 1996 | GU143522 | Bartonella spp. |
| Rattus GU143523 | *Rattus rattus brunnesculus* | Nepal | 1996 | GU143523 | Bartonella spp. |
| Rattus GU143524 | *Rattus rattus brunnesculus* | Nepal | 1996 | GU143524 | Bartonella spp. |
| Rattus GU143525 | *Rattus rattus brunnesculus* | Nepal | 1996 | GU143525 | Bartonella spp. |
| Rattus GU143526 | *Rattus rattus brunnesculus* | Nepal | 1996 | GU143526 | Bartonella spp. |
| Rattus GU143527 | *Rattus rattus brunnesculus* | Nepal | 1996 | GU143527 | Bartonella spp. |
| Rattus GU143528 | *Rattus rattus brunnesculus* | Nepal | 1996 | GU143528 | Bartonella spp. |
| Rattus GU143529 | *Rattus rattus brunnesculus* | Nepal | 1996 | GU143529 | Bartonella spp. |
| Rattus GU143545 | *Rattus rattus brunnesculus* | Nepal | 1996 | GU143545 | Bartonella spp. |
| Rattus GU143546 | *Rattus rattus brunnesculus* | Nepal | 1996 | GU143546 | Bartonella spp. |
| Rattus GU143547 | *Rattus rattus brunnesculus* | Nepal | 1996 | GU143547 | Bartonella spp. |
| Rattus GU143548 | *Rattus rattus brunnesculus* | Nepal | 1996 | GU143548 | Bartonella spp. |
| Rattus GU143549 | *Rattus rattus brunnesculus* | Nepal | 1996 | GU143549 | Bartonella spp. |
| C5RAT | *Rattus norvegicus* | Peru | 1997 | Z70018 | Bartonella spp. |
| C7RAT | *Rattus norvegicus* | Peru | 1997 | Z70020 | Bartonella spp. |
| RR13863PO | *Rattus rattus* | Portugal | 1999 | AF086636 | Bartonella spp. |
| Gs5686t_MB17 | *Grammomys sp* | Tanzania | 2008 | FJ851112 | Bartonella spp. |
| Ld5743t_MB3 | *Lophuromys dudui* | Tanzania | 2008 | FJ851109 | Bartonella spp. |
| Ld5693t_MB31 | *Lophuromys dudui* | Tanzania | 2008 | FJ851118 | Bartonella spp. |
| Ld480t_MB36 | *Lophuromys dudui* | Tanzania | 2008 | FJ851120 | Bartonella spp. |
| Ld481t_MB37 | *Lophuromys dudui* | Tanzania | 2008 | FJ851121 | Bartonella spp. |
| Ld5742t_MB11 | *Lophuromys dudui* | Tanzania | 2008 | FJ851111 | Bartonella spp. |
| Pd5708t_MB23 | *Praomys diwenyi* | Tanzania | 2008 | FJ851116 | Bartonella spp. |
| Pd5728t_MB34 | *Praomys diwenyi* | Tanzania | 2008 | FJ851119 | Bartonella spp. |
| Pd5692t_MB2 | *Praomys diwenyi* | Tanzania | 2008 | FJ851108 | Bartonella spp. |
| Pd5696t_MB6 | *Praomys diwenyi* | Tanzania | 2008 | FJ851110 | Bartonella spp. |
| Pd5695t_MB21 | *Praomys diwenyi* | Tanzania | 2008 | FJ851114 | Bartonella spp. |
| Pd5700t_MB22 | *Praomys diwenyi* | Tanzania | 2008 | FJ851115 | Bartonella spp. |
| Bi3726th | *Bandicota indica* | Thailand | 2002 | AY277891 | Bartonella spp. |
| Bi3778th | *Bandicota indica* | Thailand | 2002 | AY269417 | Bartonella spp. |
| Bi3781th | *Bandicota indica* | Thailand | 2002 | AY269418 | Bartonella spp. |
| Bi4251th | *Bandicota indica* | Thailand | 2002 | AY269421 | Bartonella spp. |
| Bi4271th | *Bandicota indica* | Thailand | 2002 | AY277893 | Bartonella spp. |
| Bi4298th | *Bandicota indica* | Thailand | 2002 | AY269419 | Bartonella spp. |
| Bi5131th | *Bandicota indica* | Thailand | 2002 | AY264493 | Bartonella spp. |
| RL3725th | *Rattus losea* | Thailand | 2002 | AY269279 | Bartonella spp. |
| RL5132th | *Rattus losea* | Thailand | 2002 | AY269420 | Bartonella spp. |
| Rr4252th | *Rattus losea* | Thailand | 2002 | AY277892 | Bartonella spp. |
| Bi8780th | *Bandicota indica* | Thailand | 2004 | FJ655392 | Bartonella spp. |
| Bi8781th | *Bandicota indica* | Thailand | 2004 | FJ655393 | Bartonella spp. |
| Bi8769th | *Bandicota indica* | Thailand | 2004 | FJ655394 | Bartonella spp. |
| Bs19435th | *Bandicota saviley* | Thailand | 2004 | FJ655395 | Bartonella spp. |
| Bs19298th | *Bandicota saviley* | Thailand | 2004 | FJ668633 | Bartonella spp. |
| Rr18694th | *Rattus rattus* | Thailand | 2004 | FJ655399 | Bartonella spp. |
| Rr18771th | *Rattus rattus* | Thailand | 2004 | FJ655400 | Bartonella spp. |
| Rr19309th | *Rattus rattus* | Thailand | 2004 | FJ655401 | Bartonella spp. |
| Ra19426th | *Rattus argentiventis* | Thailand | 2004 | FJ655402 | Bartonella spp. |
| Rb19945th | *Rattus berdmorei* | Thailand | 2004 | FJ655403 | Bartonella spp. |
| Re19170th | *Rattus exulans* | Thailand | 2004 | FJ655404 | Bartonella spp. |
| Rr19140th | *Rattus rattus* | Thailand | 2004 | FJ655405 | Bartonella spp. |
| Rr19162th | *Rattus rattus* | Thailand | 2004 | FJ655406 | Bartonella spp. |
| Rr18720th | *Rattus rattus* | Thailand | 2004 | FJ655407 | Bartonella spp. |
| Rr18774th | *Rattus rattus* | Thailand | 2004 | FJ655408 | Bartonella spp. |
| Rr19192th | *Rattus rattus* | Thailand | 2004 | FJ655409 | Bartonella spp. |
| Rr19247th | *Rattus rattus* | Thailand | 2004 | FJ655410 | Bartonella spp. |
| Rr19248th | *Rattus rattus* | Thailand | 2004 | FJ655411 | Bartonella spp. |
| Rr19306th | *Rattus rattus* | Thailand | 2004 | FJ655412 | Bartonella spp. |
| Rr19360th | *Rattus rattus* | Thailand | 2004 | FJ655413 | Bartonella spp. |
| Rr19363th | *Rattus rattus* | Thailand | 2004 | FJ655414 | Bartonella spp. |
| Rr19370th | *Rattus rattus* | Thailand | 2004 | FJ655415 | Bartonella spp. |
| Tabanzu R rattus 106 REV | *Rattus rattus* | Uganda | 2011 | KC763960 | Bartonella spp. |
| Jupakonja R rattus 255 REV | *Rattus rattus* | Uganda | 2011 | KC763968 | Bartonella spp. |
| RR11755TX | *Rattus rattus* | USA | 1999 | AF075167 | Bartonella spp. |
| Rn10149MD | *Rattus norvegicus* | USA | 1998 | AF075161 | Bartonella spp. |
| Rn10616LA | *Rattus norvegicus* | USA | 1998 | AF075162 | Bartonella spp. |
| Rn10617LA | *Rattus norvegicus* | USA | 1998 | AF075163 | Bartonella spp. |
| Rn10623LA | *Rattus norvegicus* | USA | 1998 | AF075164 | Bartonella spp. |
| Rn10627LA | *Rattus norvegicus* | USA | 1998 | AF075165 | Bartonella spp. |
| Rn10631LA | *Rattus norvegicus* | USA | 1998 | AF075166 | Bartonella spp. |
| Rattus B27363-R234 | *Rattus rattus* | USA | 2009 | KC763949 | Bartonella spp. |
| Rattus B27364-R256 | *Rattus rattus* | USA | 2009 | KC763949 | Bartonella spp. |
| Rattus B27366-R264 | *Rattus rattus* | USA | 2009 | KC763949 | Bartonella spp. |
| Rattus B27367-R273 | *Rattus rattus* | USA | 2009 | KC763949 | Bartonella spp. |
| Rattus B27368-R274 | *Rattus rattus* | USA | 2009 | KC763949 | Bartonella spp. |
| SF9-246 | *Rattus rattus* | USA | 2009 | JF429451 | Bartonella spp. |
| SF9-247 | *Rattus rattus* | USA | 2009 | JF429451 | Bartonella spp. |
| SF9-252 | *Rattus rattus* | USA | 2009 | JF429451 | Bartonella spp. |
| Rn03-106LAgltA | *Rattus norvegicus* | USA | 2000 | JF429450 | Bartonella spp. |
| Rn03-108LAgltA | *Rattus norvegicus* | USA | 2000 | JF429451 | Bartonella spp. |
| Rn03-307LAgltA | *Rattus norvegicus* | USA | 2000 | KC763944 | Bartonella spp. |
| Rn03-417LAgltA | *Rattus norvegicus* | USA | 2000 | KC763945 | Bartonella spp. |
| Rn04-135LAgltA | *Rattus norvegicus* | USA | 2000 | KC763946 | Bartonella spp. |
| Rn04-272LAgltA | *Rattus norvegicus* | USA | 2000 | KC763947 | Bartonella spp. |
| Rn04-318LAgltA | *Rattus norvegicus* | USA | 2000 | KC763948 | Bartonella spp. |
| Rn04-327LAgltA | *Rattus norvegicus* | USA | 2000 | KC763950 | Bartonella spp. |
| Rn04-424LAgltA | *Rattus norvegicus* | USA | 2000 | KC763951 | Bartonella spp. |
| Rn06-121LAgltA | *Rattus norvegicus* | USA | 2000 | KC763953 | Bartonella spp. |
| Rn06-178LAgltA | *Rattus norvegicus* | USA | 2000 | KC763954 | Bartonella spp. |
| Rn06-179LAgltA | *Rattus norvegicus* | USA | 2000 | KC763955 | Bartonella spp. |
| Rn06-181LAgltA | *Rattus norvegicus* | USA | 2000 | KC763956 | Bartonella spp. |
| Rn06-183LAgltA | *Rattus norvegicus* | USA | 2000 | KC763957 | Bartonella spp. |
| Rn06-36LAgltA | *Rattus norvegicus* | USA | 2000 | KC763952 | Bartonella spp. |
| Rn06-428LAgltA | *Rattus norvegicus* | USA | 2000 | KC763961 | Bartonella spp. |
| Re6071vi | *Rattus exulans* | Vietnam | 2003 | Z70020 | Bartonella spp. |
| Re6098vi | *Rattus exulans* | Vietnam | 2003 | KC763943 | Bartonella spp. |
| Re6126vi | *Rattus exulans* | Vietnam | 2003 | KC763943 | Bartonella spp. |
| Rn6123vi | *Rattus norvegicus* | Vietnam | 2003 | FJ655404 | Bartonella spp. |
| Rn6124vi | *Rattus norvegicus* | Vietnam | 2003 | KC763959 | Bartonella spp. |
| B218RnF | *Rattus norvegicus* | Vietnam | 2008 | KC763933 | Bartonella spp. |
| B219RnF | *Rattus norvegicus* | Vietnam | 2008 | KC763934 | Bartonella spp. |
| B221RnF | *Rattus norvegicus* | Vietnam | 2008 | KC763935 | Bartonella spp. |
| B222RnF | *Rattus norvegicus* | Vietnam | 2008 | KC763935 | Bartonella spp. |
| B224RnF | *Rattus norvegicus* | Vietnam | 2008 | KC763936 | Bartonella spp. |
| B225RnF | *Rattus norvegicus* | Vietnam | 2008 | KC763937 | Bartonella spp. |
| B230RnF | *Rattus norvegicus* | Vietnam | 2008 | KC763938 | Bartonella spp. |
| B237RnF | *Rattus norvegicus* | Vietnam | 2008 | KC763939 | Bartonella spp. |
| B245RtF | *Rattus tanezumi* | Vietnam | 2008 | KC763940 | Bartonella spp. |
| B251RnF | *Rattus tanezumi* | Vietnam | 2008 | KC763941 | Bartonella spp. |
| B280ReF | *Rattus exulans* | Vietnam | 2008 | KC763942 | Bartonella spp. |
| Sm6145vi | *Suncus murinus* | Vietnam | 2003 | GU143526 | Bartonella spp. |
